# Factors Influencing the Reproductive Ability of Male Bees: Current Knowledge and Further Directions

**DOI:** 10.3390/insects12060529

**Published:** 2021-06-07

**Authors:** Huiyue Zhao, Shibonage K. Mashilingi, Yanjie Liu, Jiandong An

**Affiliations:** Key Laboratory for Insect-Pollinator Biology of the Ministry of Agriculture, Institute of Apicultural Research, Chinese Academy of Agricultural Science, Beijing 100093, China; zhaohuiyue1124@163.com (H.Z.); kulindwasm@gmail.com (S.K.M.)

**Keywords:** bumblebees, honeybees, male reproductive ability, environmental factors, internal factors

## Abstract

**Simple Summary:**

Bumblebees and honeybees are well known as the dominant and most important pollinators in natural and agricultural ecosystems. The quality characteristics of their colonies depend greatly on the reproductive ability/quality of the parents (queens and drones). Male bees, despite their exclusive reproductive role and ability to determine colony quality, have been less considered than female bees, especially bumblebees. We reviewed the current studies on environmental factors and inherent characteristics that affect the mating success and fecundity of male honeybees and bumblebees. Temperature, nutrients, pesticides, body size, weight and age affect reproduction in male bees and consequently the progeny colony quality. However, more studies, especially in male bumblebees, are still needed to address the impacts of these factors in detail to confront the requirements of agricultural pollination and declining wild bee pollinators worldwide.

**Abstract:**

Bumblebees and honeybees are very important pollinators and play a vital role in agricultural and natural ecosystems. The quality of their colonies is determined by the queens and the reproductive drones of mother colonies, and mated drones transmit semen, including half of the genetic materials, to queens and enhance their fertility. Therefore, factors affecting drone fecundity will also directly affect progeny at the colony level. Here, we review environmental and bee-related factors that are closely related to drone reproductive ability. The environmental factors that mainly affect the sperm count and the viability of males include temperature, nutrients and pesticides. In addition, the inherent characteristics of male bees, such as body size, weight, age, seminal fluid proteins and proteins of the spermathecal fluid, contribute to mating success, sperm quality during long-term storage in the spermathecae and the reproductive behaviors of queens. Based on the results of previous studies, we also suggest that the effects of somatotype dimorphism in bumblebee males on sperm quality and queen fecundity and the indispensable and exploitable function of gland proteins in the fecundity of males and queens should be given more attention in further studies.

## 1. Life and Role of Male Bees in Colonies

Honeybees and bumblebees are the most important pollinators in agricultural systems. Similar to other eusocial species in the order Hymenoptera, honeybees and bumblebees live in large colonies containing a queen, workers, and males [1,2]. The nests and life cycles of the two bee taxa differ in some ways. Honeybee colonies are perennial, consisting of workers and drones numbering in the thousands and a healthy fertilized queen that can live for up to five years [1]. Bumblebees are seasonal with an annual life cycle, with a few hundred workers and males composing a colony, and in some species, a population peak of over 1000 bees has been documented [2,3].

Males mainly hatch from haploid unfertilized eggs, while females (workers and queens) develop from diploid fertilized eggs [1,2]. Diploid males are also produced in both honeybees and bumblebees, and their production may pose an increased risk to the survival of the bee colonies [4,5]. The laying of male-destined eggs and the production of males are normally initiated during the reproductive season, when a sufficient population of attending workers and food resources are available [1]. Development from egg to adult differs among species, taking a relatively long time in bumblebees. On average, development in bumblebee males takes 24–28 days [6], while development takes approximately 24 days in male honeybees [1,7]. Several factors, such as the overall colony condition, temperature (brood nest), number of workers, and quality and availability of food resources (nutrients), have a crucial influence on the development time and quality of the male bumblebees produced [2].

The primary role of males in bee colonies is mating, which is a fundamental reason why they are produced only when needed. Their reproductive ability begins and completes development at early stages before emergence (larval and pupal stages). After emergence, males gradually mature to copulate and transmit sperm to the spermathecae of queens. Estimates of sexual maturation range between 6–20 days after emergence in bumblebees [6] and 6–16 days in honeybees [7]. Efficient copulation and sperm viability are closely related to male quality characteristics such as birth weight, age, body size, and environmental conditions. Apart from their exclusive reproductive role, male bees contribute to heat regulation in the colony, although their contribution is no more than that of worker bees [8].

Male honeybees are unable to engage in foraging behavior as they lack food collection body parts [9]. Usually, during the dearth of food or at the end of the reproductive season, the laying of male-destined eggs halts, and adult males are no longer needed and are thus forcefully evicted from the colony [1,9,10]. Other factors have also been suggested to contribute to the eviction of drones, e.g., reduced environmental temperature, the presence of workers with developed ovaries, the presence of a queen, the age of the queen, the amount of sealed and unsealed brood, the activity of the colony, the amount of forage being collected, the amount and condition of honey stores, and the strain of the bees [10]. Evicted males starve since they lack food collection body parts such as a honey stomach and pollen basket for foraging [1,9]. In contrast, multiple studies have documented the foraging and pollination abilities of male bumblebees [11,12,13,14,15]. Mature male bumblebees leave their nest in search of virgin queens with which to mate and engage in foraging and flower pollination [13,15]. Fliszkiewicz and colleagues noted the pollination effect of male *Bombus terrestris* to be similar to that of worker bees [14], and when trained to identify fodder color, freely flying male *B. terrestris* behave as workers in terms of learning speed and accuracy [12]. However, they need to make a trade-off between foraging and mating with queens. Male bumblebees can also act as an excellent alternative model system for further studies of pollination capacity due to their ability to successfully pollinate some crops, such as the blackcurrant *Ribes nigrum* and *Gentiana parryi* (Gentianaceae) [13,14,15].

## 2. Reproductive Ability of Male Bees

Mating behavior is an aspect of the reproductive ability of male bees that influences the offspring of the colony. Although successful mating is fatal for male honeybees, honeybee queens can mate with multiple males to store sufficient sperm. This possibility of nonvirgin queens mating again is unfavorable for *Apis mellifera* drones in terms of passing on their genes [16,17]. To prevent the mated queens from flying out of the colonies to mate again, male semen changes the expression of genes associated with vision in the queens’ brains, weakening the reaction of their compound eyes and ocelli to light. This makes it difficult for a mated queen that has flown to mate again to return to the colony, causing her to face the risk of death [18]. The selective pressure of sperm competition may cause the production of higher-quality sperm in male honeybees than those monandrous bumblebee species [19]. In contrast to the fatal fate of male honeybees after successful mating, male bumblebees are able to mate multiple times. Moreover, the mating frequency of queens varies widely between different species of bumblebees, for instance, the queens of *B. terrestris*, *B. lantschouensis*, *B. patagiatus* and *B. ignitus* mate only once in their lifetime, while queens of *B. hypnorum* can mate up to six times [20,21]. Queens that mate with nonvirgin males can produce more workers and males and are thus more successful in building colonies [22]. Multiple mating may be closely related to the length of sperm and the selection for longer sperm, which can be related to body size [23]. Male bumblebees prevent the sperm of other males from entering the queen’s genital tract with the placement of a mating plug that reduces sperm competition. For instance, in *B. terrestris*, the ubiquitous and rather unspecific fatty acid linoleic acid composition of the male mating plug prevents females (queens) from further mating [24]. Male characteristics such as age and weight influence the duration of copulation and mating success. A short copulation duration could delay the establishment of colonies, although it has been found to have no impact on the production of new queens and males [25]. Environmental factors that may affect the mating process have been described in previous reviews, such as the temperature of the hive and drone nutrients [11,26]. Furthermore, the mating rate differs in different species and has been found to be affected by internal factors in both bumblebees and honeybees. In this regard, there might be some unique factors that control the mating behavior of males of different bumblebee species, which is very important to the knowledge of social insects.

**Figure 1:**
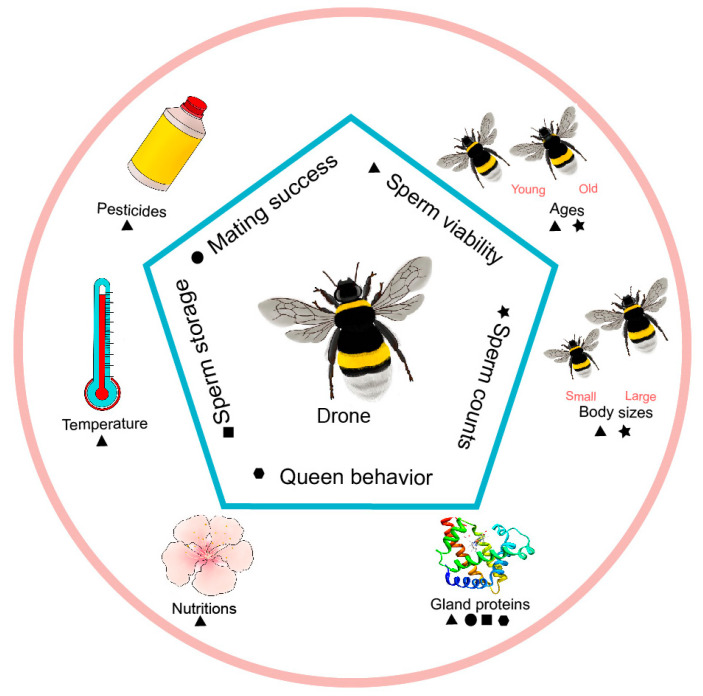
The main aspects of the reproductive ability of male bees are affected by various environmental factors and inherent characteristics, including ▲ sperm viability, ★ sperm counts, ⬣ queen behavior, ■ sperm storage, ● mating success.

In addition to mating behavior, sperm quality is a key factor influencing successful reproduction in bee colonies since semen will be stored for more than three years in the spermathecae of honeybee queens [1] and for several months in the spermathecae of bumblebee queens after insemination. Several parameters have been used to evaluate the reproductive ability of male bees, such as sperm motility, sperm viability, and acrosomal integrity. Sperm motility is important for the transfer of sperm into the queen’s spermathecae [27] and has been used as an essential indicator for estimating sperm quality [28,29]. Sperm viability is closely linked to the fertility of queens and is thus widely assayed to evaluate semen quality [30,31]. However, sperm quality is irrelevant to queen longevity, which is associated with the mating behavior or substances received from mating in eusocial insects such as ants [32]. However, sperm influence the hibernation success and fitness of inseminated females, and sperm of different male genotypes show distinct female longevity in generally monandrous *B. terrestris* [33,34]. Most studies related to the cryopreservation of frozen semen regard acrosomal integrity as an indicator of digestion of the zona pellucida and the fusion ability of sperm [35,36]. Yaniz and colleagues summarized most of the parameters and several kinds of methods for the analysis of semen quality in honeybees [37]. Poor male reproductive ability may affect health and productivity by influencing queen fecundity, which determines subsequent worker behavioral or physiological traits and is influenced by semen fluid and viable sperm [38]. Moreover, multiple mating increases the genetic diversity of sperm in queens, swarms headed by which show great advantages in the speed of founding new colonies, foraging rates, food storage, drone population, winter survival and pathogen resistance [39,40,41]. In the current paper, we review the factors affecting sperm quality in ejaculate and spermathecae in both honeybees and bumblebees (Figure 1).

## 3. Influence of External Environmental Factors on the Sperm Quality of Drones

### 3.1. Nutrients

Nutrients have been proven to be important for improving the reproductive quality of drones. Nectar and pollen are the prime nutrient resources used for adult and larval development in bees, and the nutritional requirements of different bees, such as honeybees and bumblebees, exhibit preferences [42,43]. Limited access to pollen during larval development in honeybee drones results in less semen and an increased likelihood of ejaculation failure [44]. The pollen species composition is a very important factor that greatly influences the population of bee colonies [45]. A colony of *B. terriestris* fed pollen with a high protein content produces the highest number of males compared to those fed pollen with a low protein content [46]. When colonies were fed sucrose syrup and protein supplements, the weight and abdominal size of the drones increased significantly. In contrast, the sperm volume and motility of each mature drone declined significantly in the colonies that received no additional nutrients [47]. A lack of protein intake after the emergence of drones does not affect sperm motility, indicating that sperm development is completed before emergence and that sexually mature drones do not need additional protein [48]. Pelletier and McNeil found that in bumblebees, food supplementation can increase the number of males and the probability of producing gynes (young queens) [49]. In addition, the bumblebee adult body size depends on the amount of food received [50,51]. Relatively large males have an advantage in male–male competition in some species [52,53,54].

The season also shows an obvious effect on the semen volume and sperm number. The maximum number of honeybee drone broods are produced in June and July [55]. Drones release significantly higher semen volumes in spring than in summer and autumn. However, autumn-reared drones produce more sperm than summer- and spring-reared drones, which produce the least sperm [56]. Seasonal factors influence drone production, including the day length and temperature, size of the colonies and availability of food [57].

However, fewer studies have addressed the effects of nutrients and seasons on the quality of sperm in bumblebees than in honeybees. Based on the species-specific characteristics of reproduction in bumblebees, more detailed and explicit studies on the influence of the nutrient dose, nutrient composition, nutrient ratio and feeding time point on drone quality attributes, such as the sperm count and viability, should be performed among different species in future research.

### 3.2. Temperature

*A. mellifera* drones incubated at the capped brood stage at a relatively low temperature (32–35 °C) deliver less semen but have higher sperm viability [58]. In the process of the in vitro storage of drone sperm, extreme temperatures can significantly reduce the viability of stored sperm. Preserving the fresh ejaculate of *A. mellifera* at high or low temperatures resulted in a significant increase in dead sperm by up to 40% [59,60]. Moreover, temperatures are variable during *A. mellifera* young queen shipment, and both low and high temperatures significantly decrease sperm viability [61]. Exposure to high-temperature extremes (45 °C) can cause more than 50% of sperm to die in the spermathecae of queens [59]. The optimal temperature (30–35 °C) significantly increases the number of living sperm after insemination [59]. Thus, the optimal temperature is a critical environmental factor affecting the maintenance of the sperm quality of drones and the storage of sperm in vitro at certain times.

A study of drones of twenty-one bumblebee species showed that male bumblebees tend to evolve larger body sizes in locations where rain occurs, mostly during summer, when the overall temperature is warmer [3]. The male size can directly impact the *B. terrestris* mating success [62]. In comparison to honeybees, bumblebees exhibit greater species diversity and occur over more complex environments and thus serve as a good model for studying the influence of climate change on bees and other insects [63]. Studies on the impact of high temperatures on the reproductive ability of bumblebees are crucial for the evaluation of the threat of global warming to insect colonies. Moreover, for bumblebee queens that live alone to pass through diapause at cold temperatures, the effects of cold temperatures on the storage of sperm in queens must be evaluated because they have important influences on the reproduction and protection of bumblebees.

### 3.3. Pesticides

Widely used pesticides have caused controversy due to their adverse effects on bee health, including those related to sperm viability in male bees and the storage of sperm in queen bees. Neonicotinoids, which are registered for use on over 140 crops, are the most widely used pesticides in more than 120 countries [64]. The neonicotinoids imidacloprid and thiamethoxam have significantly negative effects on the sperm quality of male bees. After exposure to thiamethoxam at a concentration of 4.5 ppb, the life span of male bees was significantly shortened, and the number of living sperm decreased by 39% [65]. After being exposed to an extremely high concentration of imidacloprid (0.02 ppm), the sperm motility of drones significantly decreased, although this value significantly varied among different colonies [29,66]. When bee colonies were exposed to neonicotinoid pesticides, the asymmetry of forewing veins and wing morphology fluctuation significantly increased in honeybee drones. The honeybee queen spermatheca is also highly susceptible to pesticides. Antioxidant genes in the spermatheca are inhibited after treatment with a low dose of coumaphos (5 ppm) and a sublethal dose of imidacloprid (0.02 ppm) [67]. Exposure to a sublethal dose of imidacloprid (0.02 ppm) or a high dose of coumaphos (100 ppm) can result in a decline in sperm viability by 50% or 33% in the spermatheca, respectively [67].

Another common type of chemical exposure is exposure to pesticides used for bee disease prevention by beekeepers, such as amitraz, which is used to control *Varroa destructor*, a major pest of honeybees. Sublethal doses of amitraz had no effect on the viability of sperm stored in honeybee queens under laboratory conditions or sperm viability. However, it affected development and downregulated genes related to detoxification, cyclic adenosine monophosphate (cAMP)-dependent protein kinase, immunity, and antioxidant capacity [68].

In addition, fipronil syrup is a phenylpyrazole insecticide. After eating it at a concentration of 0.1 mg/L, the sperm concentration and sperm motility of honeybee drones were found to decrease significantly, and the sperm metabolic rate increased, leading to reduced fertility in the drones [69]. Many moderately toxic and low-toxicity pesticides, such as fungicides and herbicides, also have lethal and sublethal impacts on bees [6].

Overall, research studies on the effects of pesticides on bees are mainly conducted in Europe and North America, assessed in honeybees under laboratory conditions, and measured at the individual level [70]. Low doses or nonlethal doses of diverse pesticides might not cause the death of bees but may decrease some of their abilities, such as reproduction. Therefore, we suggest that studies should combine the effects on individual bees and on bee colonies of more bee species to discuss the toxicity of neonicotinoids at the population level and how the effects pose a threat to the reproductive ability of male bees. The effects of low or nonlethal doses of diverse pesticides on the sperm quality and foraging behavior of male bees should be evaluated in detail. Moreover, some insecticides used by beekeepers to prevent bee diseases should be assessed further in future studies, evaluating their influence on the sperm quality of drones and the storage of sperm in queens.

## 4. Influence of the Internal Environment on the Sperm Quality of Drones

### 4.1. Age and Birth Weight

Several studies have reported that honeybee sperm production, maturation and viability are closely related to age. Hölldobler and Bartz reported that sperm production in social insect males ceases upon eclosion [71]. The honeybee drone also ecloses with all the sperm [72], and then the sperm move from the testes to the seminal vesicles, during which the sperm or seminal fluid gradually matures with age (6–14 days after eclosion). The semen volume in the seminal vesicles gradually decreases, the sperm density increases, and the composition of the ejaculate changes [73]. The age of drones displays a continuing effect on the semen volume and sperm concentration. For instance, 14-day-old and 21-day-old drones release more semen volume in the ejaculate than 35-day-old drones, and 21-day-old drones release higher sperm counts in the ejaculate than 14-day-old and 35-day-old drones that share similar sperm numbers [56]. Ruttner and Tarpy noted that the sperm counts in the seminal vesicles reached 7,390,000 at 20 days after emergence and decreased significantly 30 days later [74]. In addition to the semen volume and sperm counts, sperm viability is closely correlated with drone age. Locke and Peng reported that sperm viability decreased significantly with increasing drone age, while motility patterns did not change [75]. Sturup found that the sperm viability of *A. mellifera* decreases with drone age, with differences among colonies, some of which could delay ejaculation senescence [48]. However, recent studies show that drone age and time of breeding have no effect on sperm viability [74,76]. Moreover, sperm DNA fragmentation and the coagulation and proteolysis of semen are closely related to drone age and have notable effects on sperm. The coagulation of semen occurs in old drones, and these sperm display the lowest sperm surface protease activities, with both characteristics negatively affecting sperm viability [77,78]. The correlation between sperm DNA fragmentation and male age is controversial in animals based on current studies and exhibits a significantly negative effect on male fertility [79]. Unfortunately, studies paid scant attention to the sperm DNA fragmentation alongside drone age in bees. Currently, we only know that 14-day-old honeybee drones possess significantly higher DNA fragmentation rates in sperm than those collected directly from queens of different ages [80], hence, it is meaningful to explore the DNA fragmentation rate in male bees from different age and the connection with sperm quality. Studies in the bumblebee *B. terrestris* have noted that age and weight influence the duration of copulation. Younger males copulated more quickly and copulated for a shorter duration than old males, and relatively heavy males of *B. terrestris* copulated more rapidly than lighter males [62]. However, studies on the relationships between the age, birth weight, and sperm viability of males are currently lacking for bumblebees. The ages of males and queens are critical to mating success and the development of offspring colonies in bumblebees.

### 4.2. Body Size

Czekonska reported that the weight of honeybee drones at birth is an important factor in predicting the survival of drones in colonies and that males that were heavier at birth reached sexual maturity 15 days after emergence and lived longer than males that were lighter at birth [81]. In queenright colonies (QRCs) of honeybees where the queens are in charge, reproductive workers produce only 7% of the unfertilized eggs in the colony. When the queen is lost, the expression level of vitellogenin in nonreproductive workers increases, and workers from different subfamilies of honeybees all tend to lay eggs with different success rates [82,83]. Drones produced in the drone cells (DCs) of QRCs are 17% heavier than those produced in the DCs of laying worker colonies (LWCs), while drones produced in worker cells (WCs) in LWCs have the smallest body size and lightest weight [84]. The length of the wing is thought to be an effective indicator for distinguishing large drones (LDs) from small drones (SDs) since the length and width of the front wing of LDs are significantly larger than those of SDs [85]. Body size is closely related to the sperm count of male bees. The average sperm count and accessory mucous gland and seminal vesicle weights of LDs were found to be significantly higher than those of SDs [84,86]. The SDs are only 87% of those of normal drones on average, and the sperm count is also significantly lower than that of normal-size drones [87].

Moreover, body size also affects the mating success of male bees. Couvillon and colleagues found that within a day, the flight activity of LDs peaked at noon, which is consistent with the peak in queen activity, while the flight activity of SDs was more uniform throughout the day [88]. Gençer and Kahya quantified the sperm competitiveness of SDs reared in laying worker colonies (LWCs) against LDs reared in QRCs and found that SDs were not as successful as LDs in terms of sperm competition. Gencer and Kahya mixed the sperm of two types of male bees of *A. mellifera* in different proportions for artificial insemination of queens. The paternal frequencies in the offspring of SDs were all lower than expected, while large males produced more offspring than expected [89]. Although SDs remained slightly behind LDs in regard to sperm competition, this does not imply that SDs reared in LWCs are useless. In addition, the concentration of sperm in the ejaculate affects the reproductive success of drones, and evolution forces drones to produce not only plentiful but also particularly concentrated semen [89].

The somatotype dimorphism of males is more obvious in bumblebees than in honeybees. Bumblebee workers are also capable of producing male offspring from unfertilized, haploid eggs, but parentage analyses suggest that only a low proportion (approximately 5%) of adult males are worker produced [90,91]. Lacking queen-specific chemical cues, worker-laid eggs can be distinguished and quickly cleared by other workers in the bumblebee *B. terrestris* [92,93]. An observation in our lab showed that males from the QRCs of *B. terrestris* have a distinctly larger body size than that produced by the workers (unpublished data). Large males may have relatively large genitalia that act as particularly strong forceps for attachment to the queen’s sting apparatus [94,95]. Large/young drones initiate mating more quickly and copulate for less time than lighter, older drones. The lengths of the front and hind feet affect mating success as age becomes less important in competitive situations, such that drones with longer feet have higher mating success than drones with shorter feet [62]. Moreover, males from a queenright bumblebee colony also exhibited diverse body sizes. Sperm length was found to be positively correlated with male body size in *B. terrestris* and possibly in *B. hypnorum* [23]. Therefore, somatotype dimorphism in male bees, especially bumblebees, can best explain the influence of drone size on the colony. However, to the best of our knowledge, no research explains its influence on sperm quality and queen fecundity. Body size is an important factor for breeding queens in both bumblebees and honeybees and should be given more attention in further research.

### 4.3. Gland Proteins

Male insect semen contains sperm cells and seminal fluid components [96,97,98,99,100]. Seminal fluid is composed of proteins and other small molecules, including peptides, sugars, and lipids and is primarily derived from the male accessory glands [96,101]. Furthermore, how seminal fluid and seminal fluid proteins (SFPs) influence male and female fertility and behavior, sperm viability, and susceptibility has been intensively studied for decades in *Drosophila*. Proteins from the accessory glands of *D. melanogaster* males have been proven to be an essential factor influencing the transfer of sperm and play an important role in the storage of sperm in females. For example, accessory gland proteins are needed for morphological changes in the female reproductive tract that may be important for sperm storage [102]. Seminal fluids have been studied in other insects, including mosquitos, crickets, ants, moths, and beetles [103,104,105,106,107,108,109,110,111,112,113].

In honeybees, the activity of sperm in ejaculated semen is usually higher than that of sperm stored in the spermathecae, which benefit from the proteins in the semen [114]. Greeff found that the proportion of dead sperm in the testes of bumblebee drones was significantly lower than that in the spermathecae of newly mated queens and old queens [115]. The higher sperm viability might result from the components of the semen. The semen of *A. mellifera* is so effective at keeping sperm alive that the positive effects can last up to 24 h. The presence of proteins in semen and their structural integrity are critical to this effect, which cannot be replicated by ordinary protein substitutes [116]. Moreover, semen fluids are largely responsible for stimulating postmating changes in queen behavior and physiology, e.g., reduced sexual receptivity, reduced attraction to light, reduced mating flight, ovary activation, ovulation, modulation of pheromone production and transcriptional changes in queens [117,118]. Honeybee drone seminal fluid could induce a decline in queen vision by causing substantial gene expression changes in the brain and perturbing the phototransduction pathway to reduce queen promiscuity across mating flights [18]. This regulatory mechanism is comparable to earlier findings in *Drosophila*, with changes triggered by the sex peptide [119]. Similar changes in gene expression of the phototransduction pathway, neuroactive ligand-receptor interactions, the Hippo signaling pathway and the phagosome pathway were found in the postmating queens of *B. terrestris* [120]. Mass spectrometry has been used to identify the proteins in honeybee drone semen [99], accessory gland-associated proteins [99], seminal fluid [96,121,122,123,124], and sperm cell-associated proteins [114]. Approximately 260 proteins have been identified in honeybee seminal fluid [122]. Proteins in the accessory mucous gland of drones are the main component of semen, and the proteins in accessory glandular fluid significantly improve sperm motility [125]. Further research on *Drosophila* has identified some specific proteins that reduce female sexual receptivity and benefit rival sperm (i.e., sex peptide) [126], maintain sperm viability (i.e., Acp29AB), promote uterine contractions (i.e., Acp36DE), and promote ovulation (i.e., ovulin [127]). Interestingly, homologs of these specific proteins have not been identified within the honeybee genome, underscoring the uniqueness of the honeybee mating system and the need for specific investigations into this system [38]. Furthermore, the homologs of these specific proteins have not been identified in known bumblebee genomes that exhibit some distinctions from the honeybee genome.

In addition, both bumblebee and honeybee queens have a special organ, the spermatheca, for the long-term storage of sperm throughout their lifetime. This is especially true for most bumblebee species, which must experience a diapause period at cold temperatures. Insect spermathecae have associated secretory cells (spermathecal secretory cells or SSCs) that produce proteins and other molecules that function in sperm storage [128,129,130]. Female reproductive fluid (FRF), which significantly affects sperm traits, including chemoattraction and alterations in sperm velocity, has been shown to exert positive phenotypic effects on sperm competition in males [131]. After mating in *Drosophila* [132], *Apis* [133], *Crematogaster* [134], and *Anopheles* [135], molecules involved in the immune response, carbohydrate and lipid metabolism, cellular transport and oxidative stress have been identified in the transcriptional and proteomic profiles of sperm storage organs and might play protective roles in sperm and/or mediate female postmating processes. Interestingly, the spermathecae of *A. mellifera* and *Crematogaster osakensis* ant queens also possess some important proteins that can significantly improve sperm motility and are involved in the long-term maintenance of stored sperm [125,134]. The vesicular gland is the main contributor to the proteome of the spermathecae, but there is a more complete protein network in this organ that is conducive to long-term sperm storage [96,133]. Hundreds of proteins representing the main components of spermathecal fluid have been identified. They belong to a series of different functional groups, the most obvious of which are energy metabolism enzymes and antioxidant defense enzymes [133]. The transcripts of two antioxidative enzymes, catalase and glutathione-transferase (GST), are ten to twenty times higher in the spermathecae of mating queens than in those of unmated queens, and the expression levels of the antioxidant genes TXN2 and TXNRD1 in the spermathecae of mating queens are also higher than those in the spermathecae of nonmating queens to protect the sperm from damage [105,136,137]. Furthermore, some metabolites were identified and found to be enriched in metabolic pathways, including glycerophospholipid (GPL) metabolism, the biosynthesis of amino acids, and the mTOR signaling pathway, likely contributing to the long-term maintenance and protection of sperm [138]. In summary, SFPs and gland proteins in spermathecae exhibit diverse functions and play a significant role in the reproduction of insects (Table 1). Therefore, the function of SFPs and gland proteins in spermathecae in terms of sperm quality should be studied in bumblebees and honeybees, as this information is very important for the long-term storage of sperm in vitro and the artificial regulation of the reproduction of bumblebees and honeybees in the future.

## 5. Further Research Directions

Throughout the review, knowledge of the effects of age, birth weight, and body size on sperm viability is currently lacking for bumblebees, which is important for copulation by useful drones for bumblebee breeding. Fewer studies have reported the effects of nutrients on the quality of sperm in bumblebees compared to honeybees, and this should be addressed in further research [44,47]. Furthermore, the impacts of pollen diversity and seasons on male development and sperm quality need to be clarified. Moreover, the SFPs and gland proteins in the spermathecae of bumblebees should be identified, and the functions of SFPs and gland proteins in spermathecae in terms of sperm quality in long-term storage of sperm and postmating changes of queens in bumblebees and honeybees should be studied in further research. Moreover, the mechanism by which SFPs or sperm contribute to the hibernation success of bumblebees should be given more attention. Current studies on the exposure of drones to pesticides mainly focus on the negative effect on sperm viability [66,67,68,69]; however, pesticides may change the mating behavior of drones, which is crucial to mating success. Therefore, we suggest that more research on the impacts of various pesticides on drones should cover the mating behaviors of drones exposed to pesticides.

In summary, the current studies on male bumblebees are still much less thorough than those on male honeybees. Hence, more studies should be systematically carried out on male bumblebees that differ from male honeybees in terms of biology and lifecycle. More in-depth research regarding the factors that influence male reproduction is needed in honeybees and bumblebees, which will benefit the comprehensive evaluation of male bees’ reproductive ability in bee breeding. We highlight the knowledge gaps of current studies on the reproductive ability of male bees, it is meaningful for researchers to pay close attention and conduct in-depth research to fill these knowledge gaps. More factors, such as DNA fragmentation, genetic abnormalities, mitochondrial DNA mutations, infections, and signaling mechanisms involved in sperm motility that are thought to affect the sperm quality of humans, might also need to be considered in further research.

## Figures and Tables

**Table 1:** Functions of proteins in accessory gland of male insects and in spermathecae of queen insects presented throughout the review.

Organ	Key Points	Descriptions	Species	References
Accessory gland in male	Maintenance of sperm viability	The semen is so effective at keeping sperm alive that the positive effects can last up to 24 h cannot be replicated by ordinary protein substitutes	*Apis mellifera* *Bombus terrestris*	[115,116,125]
	Contribution to sperm transfer and storage	Alter expression of several genes and change morphology of queen’s reproductive tract.	*Drosophila melanogaster*	[102]
	Sperm competition	Change the expression of genes in the queens’ brains associated with vision to restrain queens to mate again, reduce sexual receptivity. Directly benefit the previous sperm stored in females.	*Apis mellifera* *Drosophila*	[18,117,126]
	Stimulate changes of post-mating queens	Minimize the risk to sexually transmit the parasite to the queen and colony. Stimulate the release of oocytes by the ovary.	*Apis mellifera* *Drosophila melanogaster*	[121,127]
Spermathecae in queens	Maintenance of sperm viability	Higher expression of glutathione-stransferase, catalase, thioredoxin 2, and thioredoxin reductase 1 may assist in maintaining sperm viability inside the spermathecae.	*Apis mellifera*	[136]
	Facilitate long-term storage of sperm	Proteins in spermathecae fall into a range of different functional groups, most notably enzymes of energy metabolism and antioxidant defense. Seminal receptacle was enriched for genes involved in localization, signaling and ion transport. GPL metabolism, biosynthesis of amino acids, and the mTOR signaling pathway were also enriched. Mating-induced heme peroxidase (HPX) 15 as an important factor in long-term fertility.	*Apis mellifera* *Drosophila melanogaster* *Crematogaster osakensis* *Anopheles gambiae*	[132,133,134,135,137,138]
